# Evolution of Diploid Progenitor Lung Cell Applications: From Optimized Biotechnological Substrates to Potential Active Pharmaceutical Ingredients in Respiratory Tract Regenerative Medicine

**DOI:** 10.3390/cells10102526

**Published:** 2021-09-24

**Authors:** Alexis Laurent, Philippe Abdel-Sayed, Nathalie Hirt-Burri, Corinne Scaletta, Murielle Michetti, Anthony de Buys Roessingh, Wassim Raffoul, Lee Ann Applegate

**Affiliations:** 1 Regenerative Therapy Unit, Lausanne University Hospital, University of Lausanne, CH-1066 Épalinges, Switzerland; alexis.laurent@unil.ch (A.L.); philippe.abdel-sayed@chuv.ch (P.A.-S.); nathalie.burri@chuv.ch (N.H.-B.); corinne.scaletta@chuv.ch (C.S.); murielle.michetti@chuv.ch (M.M.); 2 TEC-PHARMA SA, Manufacturing Department, CH-1038 Bercher, Switzerland; 3 LAM Biotechnologies SA, Manufacturing Department, CH-1066 Épalinges, Switzerland; 4 Children and Adolescent Surgery Service, Lausanne University Hospital, University of Lausanne, CH-1011 Lausanne, Switzerland; anthony.debuys-roessingh@chuv.ch; 5 Romand Burn Center, Lausanne University Hospital, University of Lausanne, CH-1011 Lausanne, Switzerland; wassim.raffoul@chuv.ch; 6 Plastic, Reconstructive, and Hand Surgery Service, Lausanne University Hospital, University of Lausanne, CH-1011 Lausanne, Switzerland; 7 Center for Applied Biotechnology and Molecular Medicine, University of Zurich, CH-8057 Zurich, Switzerland; 8 Oxford OSCAR Suzhou Center, Oxford University, Suzhou 215123, China

**Keywords:** active pharmaceutical ingredient, cell banking, cell therapy, COVID-19, diploid progenitor cells, inflammatory lung disease, lung cells, MRC-5 cells, regenerative medicine, vaccine substrate

## Abstract

The objective of this review is to describe the evolution of lung tissue-derived diploid progenitor cell applications, ranging from historical biotechnological substrate functions for vaccine production and testing to current investigations around potential therapeutic use in respiratory tract regenerative medicine. Such cell types (e.g., MRC-5 or WI-38 sources) were extensively studied since the 1960s and have been continuously used over five decades as safe and sustainable industrial vaccine substrates. Recent research and development efforts around diploid progenitor lung cells (e.g., FE002-Lu or Walvax-2 sources) consist in qualification for potential use as optimal and renewed vaccine production substrates and, alternatively, for potential therapeutic applications in respiratory tract regenerative medicine. Potentially effective, safe, and sustainable cell therapy approaches for the management of inflammatory lung diseases or affections and related symptoms (e.g., COVID-19 patients and burn patient severe inhalation syndrome) using local homologous allogeneic cell-based or cell-derived product administrations are considered. Overall, lung tissue-derived progenitor cells isolated and produced under good manufacturing practices (GMP) may be used with high versatility. They can either act as key industrial platforms optimally conforming to specific pharmacopoeial requirements or as active pharmaceutical ingredients (API) for potentially effective promotion of lung tissue repair or regeneration.

## 1. Introduction

Vast historical experience has been gathered around industrial use of diploid progenitor cell sources (e.g., MRC-5, WI-38 cell types) as vaccine substrates since the 1960s, when developmental cell biology studies served as a basis for optimization of biological starting material selection [1,2,3,4,5,6,7]. Despite extensive use of such materials, as well as ethical and moral debates centered mainly upon the original tissue procurement, such diploid cells have been instrumental in the development and therapeutic application of several life-saving products over a half-century [8,9,10,11,12,13,14,15,16,17,18]. The increasing material demand driven by modern biotechnological industrial development and aging of the original cell sources (i.e., stability issues) has recently prompted renewed consideration for novel substrate cell type establishment and qualification [19,20,21,22]. The unique sustainability and safety aspects of diploid progenitor cell sources, exploited as safe and stable multi-tiered biobanks, are emerging as critical advantages in modern manufacturing and quality assurance environments [23,24,25,26]. Most importantly, and in addition to the considerable inherent technical advantages of such cellular materials, a vast therapeutic potential exists for the use of diploid progenitor cells or cell derivatives for application in allogeneic regenerative medicine [27,28,29,30,31,32]. Indeed, skin-derived diploid progenitor cell sources have been extensively studied and clinically applied, in particular for the therapeutic management of pediatric burns and chronic inflammatory cutaneous wounds [33,34,35,36,37,38,39,40,41,42,43,44]. 

Optimized methodological aspects concerning tissue procurement and processing for progenitor cell isolation were the foundations of recent work on cutaneous and musculoskeletal cell therapies [25,31,45,46,47,48,49,50]. Based on available technical experience in diploid progenitor cell GMP manufacture upscaling and transposition, current efforts are directed toward the qualification and appropriate homologation of recently isolated cells as biotechnological substrates for optimal renewal and eventual replacement of original cell stocks [32,42]. Furthermore, therapeutic approaches for the management of inflammatory lung diseases or affections and related symptoms (e.g., COVID-19 patients and burn patient severe inhalation syndrome) using local homologous allogeneic cell-based or cell-derived product administrations are considered. 

The present narrative review compiles and discusses the potential and highly polyvalent valorization pathways to be considered for robust lung tissue-derived diploid progenitor cell sources. Such pathways comprise the recognition of specific cell sources by central health authorities as qualified and suitable vaccine production substrates or the appropriate registration of cell sources as active pharmaceutical ingredients (API) for the promotion of damaged lung tissue repair or regeneration. Overall, evolutive methodological and process-based aspects of diploid progenitor cell sourcing, cell bank establishment, and cell bank exploitation are summarized in this review. These elements are discussed in view of identifying gaps and potential optimization solutions available for the establishment of safe and sustainable biological material supply chains in modern biotechnological manufacturing processes and/or in translational regenerative medicine. 

## 2. Original Diploid Cell Type Establishment, Banking, and Uses

The inclusion of primary diploid cell sources in industrial manufacturing settings was prompted by the considerable material needs in the field of vaccine development and production during the second half of the twentieth century [51]. The generation of sufficient quantities of viral materials intrinsically relied on the availability of an effective propagation system, or “suitable substrate”, capable of appropriately maintaining successive infectious cycles. Tissue explants and fetal lung tissue-derived fibroblasts successively served to bridge the gap and allowed for industrial-scale manufacturing of diverse vaccines. However, major issues around starting material stability, sustainability, and safety created many health concerns at first and led to the development of stringent production quality and related testing requirements [51]. 

In 1929, it was demonstrated that the generation of virus material for eventual vaccine development and production was dependent upon the presence of viable host cells when tissue explants from hen kidneys were used as viral propagation tools [52]. Several decades of research and development were then necessary before the eventual replacement of animal tissue substrates by human diploid cell cultures, which became one of several standards in biotechnological manufacture for specific and quality-driven reasons [1,2,18,53]. Firstly, the successful polio vaccine trial in 1955, for which the inoculated product was developed as an attenuated vaccine, required considerable quantities of minced monkey kidney tissue (i.e., cultivated in defined 199 medium supplemented with calf serum) and depended on 1500 primates for every 10^6^ doses of vaccine [2,51]. Furthermore, it was found that some monkey kidney cells used for polio vaccines (e.g., Salk vaccine) harbored a potentially lethal virus, the SV40 simian virus, introducing a tangible iatrogenesis potential in the absence of appropriate biosafety testing and control schemes. From a regulatory standpoint, risk evaluations around the possibility that millions of American and British children were vaccinated with an SV40-contaminated Salk vaccine determined that critical material processing steps or controls should be implemented around the vaccine substrate materials [51]. Therefore, the implementation of appropriate risk-based viral biosafety testing schemes and contaminant removal or inactivation steps were considered as appropriate thereafter and up to the present, along with eventual original production substrate replacement [54,55,56,57]. 

In the 1950s and 1960s, collaborations between the Wistar Institute (i.e., Hayflick et al., Philadelphia, PA, USA) and the Karolinska Institute (i.e., Gard et al., Stockholm, Sweden) constituted an advanced platform for prenatal tissue procurement and primary cell type establishment [51]. The original purpose of such collaborations consisted in the study of developmental cell biology, yet the extensive optimization and characterization work of Leonard Hayflick soon evidenced the high stability, extensive expansion potential, and safety (i.e., tumorigenicity absence) of selected prenatal tissue-derived diploid cell types [1,2,51]. Based on such investigations, optimal human diploid cell types (e.g., WI-38, lung tissue-derived diploid cell source) were specifically developed, characterized, qualified, and proposed as safe and sustainable cell sources for vaccine production use (Table 1).

Subsequently, property rights over the cell sources of interest (i.e., WI-38 cells) constituted the object of lengthy legal disputes, with the epicenter set on Hayflick himself [51]. Indeed, at the time of the original development of this diploid cell source, Hayflick was under contract with the US government through a grant with the National Institutes of Health (NIH), which stipulated that he would title the developed cell banks back to the US government at the expiry of his mandate. However, Hayflick kept the cell banks in his possession after taking an appointment at Stanford University in 1968, creating a major legal dispute with his former employers. Such procedures were partly settled, and Hayflick further collaborated with the industry Merck in 1974 for the development of the RA 27/3 vaccine against rubella, helping to eradicate this disease through the judicious use of WI-38 cell substrates [51]. 

In parallel to the American developments of human primary diploid cell sources, similar proceedings were undertaken in 1966 by Dr. Jacobs in the United Kingdom under the auspices of the Medical Research Council (MRC). A most notable cell type was the MRC-5 source, originally isolated from lung tissue donated at 14 weeks of gestation (i.e., following medically indicated pregnancy termination in a 27-year-old psychiatric patient) [4]. The MRC-5 cell source was notably used for the development of vaccines for chickenpox, hepatitis A, polio, smallpox, and rabies. Jacobs similarly developed alternative cell sources from prenatal lung tissue (i.e., designated MRC-9), with the intention of sustainable long-term material provision for cell substrate development, production of biological products, research purposes, and diagnostic virology [7]. Most interestingly, such normal diploid prenatal lung tissue-derived cells developed in the 1960s remain in current industrial use, attesting to their high stability and sustainability, alongside more modern and manipulated cell types or cell lines [58,59,60,61]. Diploid cells have been characterized and historically qualified as robust and polyvalent cell substrates for viral propagation, as they are non-transformed and quasi-universal virus carriers, including newer respiratory SARS viruses such as H1N1 [51].

Due to the optimal qualification of human diploid cell sources as vaccine substrates and despite the considerable sustainability of such derived cell banks, a major bottleneck is currently arising with the transiently high activity in the vaccine industry and the aging of the original cell sources developed in the 1960s (e.g., MRC-5, WI-38 cell types) [3,5,20]. While optimization of product manufacturing workflows has been widely undertaken by vaccine and biologics pharmaceutical industries, relatively low attention has been set on the optimization of cell sourcing and the re-establishment of robust cell types or cell lines. Therefore, in order to avoid critical material shortages, renewal or modern establishment of appropriate starting materials for use as vaccine production substrates is currently deemed to be of utmost importance. 

## 3. Methodological and Technical Evolution since the 1960s toward Modern Cell Isolation, Culture, and Testing 

Drastic differences characterize the context in which original tissue procurement occurred in the 1960s and occurs today. They pertain mainly to quality-driven processes for material handling and ethical considerations centered on specificities of research on human subjects and donor consent. While modern technical workflows, detailed in ad hoc legal frameworks and guidance documents, may be interpreted as highly restrictive as compared to historical methods, modern safety prerequisites are critical in ensuring an acceptable overall quality level of materials serving for therapeutic product manufacture [45,46,47,48,62,63,64,65,66,67,68,69]. The specific example of the WI-38 cell source has been largely discussed, as a journalistic investigator was able to trace the original tissue donor in 2013, in order to clarify the context of the tissue donation and the notion of consent [51]. The donor indicated that no permission had been requested for the use of cells derived from donated tissues for worldwide vaccine production. Two main points of this specific case stand out when analyzed from a modern viewpoint: (i) full donor anonymity must be protected, and (ii) exhaustive consent (i.e., general and specific, covering procurement of tissues and subsequent use of derivatives) must be traceably obtained. Therefore, optimized transplantation programs provide appropriate modern platforms for well-defined and validated material procurement phases in view of cell source establishment and use [30,31]. 

**Figure 1:**
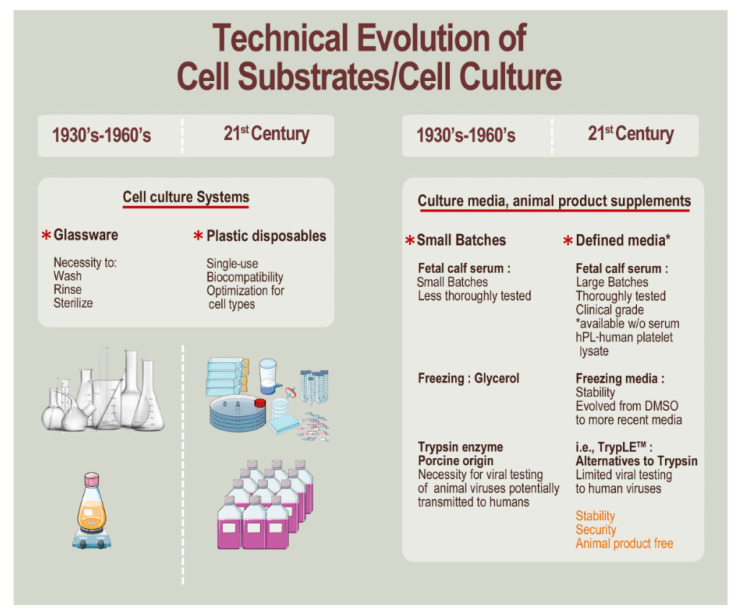
Technical evolution of cell culture conditions for vaccine substrates or biological API maintenance, processing, and storage from the 1930s–1960s era to the 21st century. Cell culture systems have evolved from cumbersome glassware requiring extensive washing, rinsing, and sterilization (i.e., trace elements impregnated in old glass) to single-use, disposable plastics that can be optimized and specifically comparatively tested for cytocompatibility. Culture growth and freezing media have evolved in terms of composition and quality, with extensive testing and qualification of larger batches and elimination of animal-sourced products, with the overall goal of increasing security and limiting biosafety-related risks for the considered manufacturing process and eventual clinical application of cell-based products. API, active pharmaceutical ingredient; DMSO, dimethyl sulfoxide; hPL, human platelet lysate.

In addition to ethical aspects of diploid cell source establishment, the evolution of cell culture methods and related tools, in particular, have contributed to the optimization of material processing and ensurance of adequate specific safety or quality levels. Notably, progress in material science has allowed for the transition from obsolete glass contact-process consumables (e.g., culture vessels, liquid handling tools) to single-use polymeric CE-marked devices, or transition from animal-sourced raw and ancillary materials (e.g., culture medium, nutrient supplements, cryopreservation medium, dissociation reagents) to defined synthetic equivalents (Figure 1) [70,71,72,73,74]. Such transitions have enabled a drastic reduction in direct manufacturing costs of cells, as well as enhanced quality and safety of manufactured cell banks or bulk product batches. 

**Figure 2:**
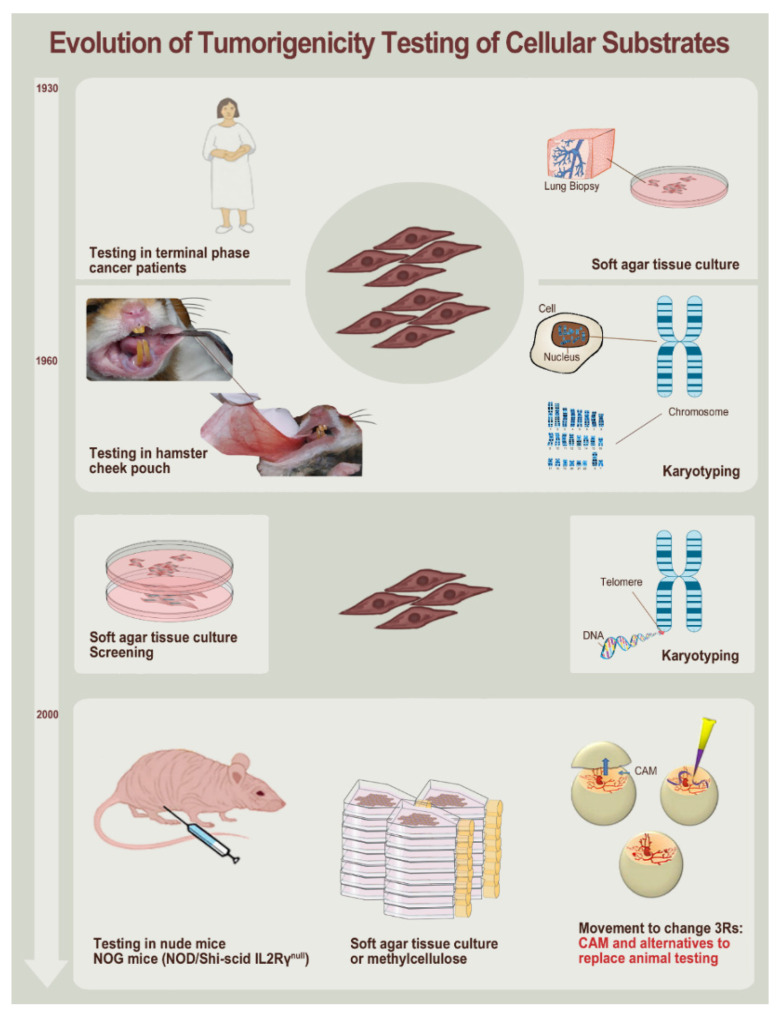
Evolution of tumorigenicity testing methodologies for cellular substrates. Testing was changed from ethically questionable use of terminal cancer patients to animal models (e.g., hamsters and mice), with the apparition of a more recent movement to avoid animal inclusion in testing processes. High throughput testing has always included soft agar cell colonization assays for preliminary screening, along with tumorigenicity testing in mice. More sensitive and alternative methods for some cell types have been implemented (e.g., in vitro use of methylcellulose), and novel mice strains have been specifically developed (e.g., NOG mice), with the designed ability to enhance the sensitivity for detection of tumorigenic potential. A current movement exists to validate alternative models to animal testing, such as the CAM model (i.e., chicken chorioallantoic membrane model), and to phase out animal experimentation for tumorigenicity testing (i.e., 3R rule for responsible animal testing: “Replace, Reduce, Refine”) [75]. CAM, chorioallantoic membrane; DNA, deoxyribonucleic acid.

A third point of interest to mention in view of illustrating the drastic differences between historic and modern processes related to cell sourcing resides in the tumorigenicity testing methodology of primary cell types, a critical parameter of cell source safety characterization and qualification. Specifically, such testing was carried out in terminal phase cancer patients in the 1930s, a practice that would be disregarded in modern medical practice (Figure 2). Thereafter, several in vitro and in vivo models were sequentially or parallelly used, such as soft agar cultures, karyotypic analyses, and test-item implantation in hamster cheek-pouches. Modern testing schemes include both in vitro and in vivo assays (e.g., embryonated eggs, NOG mice models), and subsequent optimization work may additionally be performed, notably in light of current movements toward animal experimentation rationalization (e.g., use of chorioallantoic membrane models for safety and toxicity assessment, Figure 2).

## 4. Specific Use of Diploid Cells, Derivatives, and Alternatives as Vaccine Substrates

A major discussion point with fundamental and applied moral implications around the use of diploid cells and vaccines in general has emerged, voiced notably by religious scholars in remarkable publications centered on the nature of the original biological starting material (i.e., prenatal tissues) [8,9,10,11,12,13,14,15]. Aspects ranging from cell type establishment to the use of derived vaccines by healthcare professionals are discussed, with varied viewpoints. As mentioned previously, ethical concerns with relation to the nature of starting materials should be taken into account, in particular with regard to the consideration of donor consent in the early days of diploid cell type establishment and use. Such elements are important to take into consideration for an overall assessment of specific cell sources as manufacturing tools for therapeutic products, yet current scientific and regulatory positions focus, by design, on the safety and quality of such optimized cells [50,51]. 

The main characteristics that have enabled and favored the industrial adoption of human diploid cells pertain to sustainability, stability, and robustness for application in selected manufacturing processes. Notable examples of non-transformed diploid cells, originally isolated in the 1960s from prenatal lung tissue, are the MRC-5 cell type (i.e., ATCC^®^ CCL-171™) and WI-38 cell type (i.e., ATCC^®^ CCL-75™), as mentioned previously [1,2,4]. Many vaccines (e.g., rubella, chicken pox, hepatitis A vaccines, etc.) have been continually manufactured using such cell types, despite specific under-documented aspects of original tissue procurement. Following continued industrial use, extensive characterization and qualification work, and extensive hindsight with regard to material safety, these original cell sources have come under scientific and health authority scrutiny in recent years [19,20,21,22]. Even though the specific quality of the MRC-5 cell type has been historically validated, recent concerns have been emerging around the stability, consistency, availability, and identity of this cell source [19,20,21]. Therefore, the replacement of such widely used albeit aging cell sources constitutes a modern challenge, currently exacerbated and accelerated by the manufacturing frenzy created by the worldwide COVID-19 vaccine product demand. 

In addition to non-modified human diploid cell sources, several modified cell sources and cell lines (e.g., PER.C6, HEK-293) have been developed and proposed for pharmaceutical uses toward the end of the twentieth century. The PER.C6 cell line was developed from prenatal retinoblasts (i.e., 18 weeks of gestation), which were immortalized by plasmid transfection of adenovirus type 5 (i.e., expressing R1A and elB proteins). This cell line has been primarily used for manufacturing of adenovirus vectors for gene therapy and of developmental stage vaccines for diverse pathologies caused by the Ebola virus, influenza viruses, the Japanese encephalitis virus, and HIV. A considerable technical advantage of PER.C6 cells consists in the ability to proliferate in adherent culture vessels or in suspension in serum-free media, meeting requirements for both European and US regulatory bodies [58]. Such flexibility is of key importance in pandemic situations in particular, with a capacity for rapid upscaling of manufacturing processes. However, the safety parameters of this cell line are different from those of non-modified cell sources, as they may present a tumorigenicity risk, prompting the need for the inclusion of filtration steps and cell disruption processes in the production of split and subunit vaccines.

**Figure 3:**
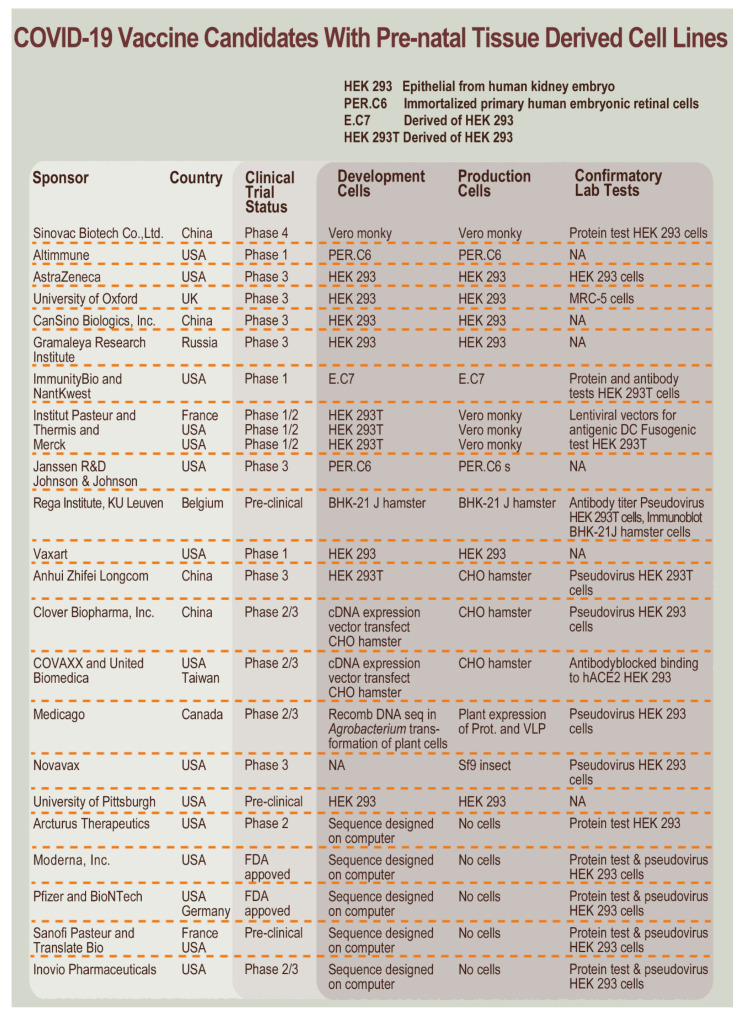
COVID-19 vaccine candidates and products for which prenatal-sourced cell types or cell lines are implicated in the development and/or production. Manufacturers use different cell sources for actual manufacturing and/or in quality and safety testing assays [76]. CHO, Chinese hamster ovary; DNA, deoxyribonucleic acid; FDA, US Food and Drug Administration; MRC, Medical Research Council; NA, not applicable; USA, United States of America.

An alternative modified cell line, the HEK-293, originally isolated from human embryonic kidney tissue, was developed and extensively used in diverse applications in medicinal product development due to a high propensity toward transfection. HEK-293 cells have been under recent public scrutiny due to the intensive use in COVID-19 vaccine development efforts (Figure 3). Indeed, although characterized as weakly tumorigenic (i.e., need for ≥10^7^ cells/test-animal to induce tumorigenicity), additional steps are also required in purification workflows if these cells are utilized in vaccine production. Specifically, vaccine manufacturers may use the HEK-293 cell line for well-defined activities in three different stages as follows:

- Development stages: to identify optimal mechanisms and processes to be used in product manufacture.
- Confirmation stage: to assure that optimal mechanisms and processes may be tangibly transposed to product manufacture.
- Production stage: to validate the use in the actual manufacturing system of the final product formula.

To provide recent examples of currently developed and approved vaccines, it is important to note that the respective Pfizer and Moderna COVID-19 vaccine developers and manufacturers used such cell lines during the development and confirmation stages but not in the actual production of the final vaccine formulation. In contrast, the Johnson and Johnson/Janssen COVID-19 vaccine was developed and is currently produced with an adenovirus carrier, where the virus vector is efficiently produced using the easily transfected PER.C6 cells. 

Alternative substrates for viral propagation are embryonated chicken eggs, which are widely used in industrial manufacturing processes. In this model, viral materials are inoculated into the allantoic cavity for propagation. This method was used since the 1940s for the propagation of many viruses, including mumps, polio, rabies, and certain veterinary vaccine viruses. Specifically, eggs provided an alternative substrate to mammalian brain tissue, which bore the potential of inducing allergic encephalitis from non-virus antigens. Furthermore, primary chicken cells/tissues were historically and importantly used for attenuated viral strain production such as yellow fever (e.g., *Flaviviridae*) and vaccinia (e.g., *Poxviridae*) [16]. Technically, the infrastructure necessary for the maintenance of eggs used in current vaccine production is held to high standards, and animal materials must notably comply with general chapter 5.2.2. “Chicken flocks free from specified pathogens for the production and quality control of vaccines” of the European Pharmacopoeia (EP), to abide by the well-defined regulatory status of specific pathogen-free animals (i.e., isolated flocks and controlled production environments, thorough biosafety screening). Intrinsic and general limitations of chicken embryo fibroblasts (e.g., limited lifespans, continuous harvests, complex logistics) introduce relatively elevated risks of contamination by adventitious agents and potential variations in permissive virus targets. Despite highly restrictive quality assurance measures for chicken embryo fibroblasts and chicken eggs in vaccine production, these substrates are used for many vaccines, including those for measles and mumps, encephalitis of ticks, rabies, yellow fever, and smallpox [18]. Overall, high necessity prompted the development of expanded strategies for egg-independent vaccine production to provide flexibility for rapid manufacture up-scaling during pandemic crises. This shift was accelerated in the context of H1N1 influenza vaccine production, when the availability delay of vaccine products (i.e., egg substrate-dependent) was 4–6 months, with potential transient bottlenecks in material supply chains (e.g., H5N1-related poultry mortality) [59]. 

A major area of investigation for optimized substrate elaboration resided in the development and use of continuous cell lines, defined as immortal cells, which maintain the karyology of the original starting tissue. While some cell lines have been immortalized by described mechanisms (e.g., transfection of PER.C6 cells), other commonly used cell lines (e.g., Vero cells) are still being further characterized for elucidation of the immortalization mechanism. Therefore, the latter may be used in medicinal product manufacturing processes, but vaccine product manufacturers need to demonstrate that the final product does not contain any oncogenic agents (i.e., a threshold of <200 bp residual DNA). Such cell lines have been historically used for inactivated poliovirus vaccine and inactivated rabies vaccine production. The Vero cells, developed from African green monkeys in 1962 (Chiba University, Chiba, Japan), presented the advantage of not being tumorigenic within certain passages, being adventitious agent-free, and could be used for the propagation of different viruses used in the manufacture of existing vaccines (e.g., polio, rota, JE, and rabies) [51]. Starting in the 1990s, several additional continuous cell sources were developed and employed in vaccine production, including human and animal tumorigenic cell lines such as HeLa (i.e., adenovirus vectors for HIV), PER.C6 (i.e., influenza and HIV), MDCK (i.e., Cocker Spaniel kidney, influenza), CHO (i.e., Chinese Hamster Ovary, HIV and Herpes Simplex Type 2), EB66 (i.e., Peking duck stem cells), insect cell lines, and other stem cell sources such as CAP (i.e., transformed human amniocytes, E1 function for adenoviral vector) [60]. More recently, the John Paul II Medical Research Institute has employed perinatal umbilical cord and placenta cell sources in COVID-19 pre-clinical work on recombinant vaccine candidates (https://rumble.com/vclsch-jp2mri-ethical-and-cutting-edge-covid-19-vaccine-research.html, accessed on 20 July 2021).

Evolutive risk-benefit analyses of continuous cell lines used as substrates, mediated by specific biotechnological advances (e.g., historical data on recombinant protein production, substrate clearance and inactivation processes), have allowed for the appropriate safety characterization and qualification of MDCK cells for influenza vaccines and PER.C6 cells in vectored vaccines. A critical parameter for cell substrate selection is the quality and safety of the finished product for the intended human therapeutic application. Therefore, residual cellular DNA in vaccine products would present a potential iatrogenic risk in the case of tumorigenic cell substrate use. Thus, it is necessary to determine the presence and quantity of residual substrate DNA (i.e., in relation to specified limits of content and DNA strand length) in a quantitative assay to measure both infectivity and oncogenic activities of said residues [61].

## 5. Renewal of Vaccine Substrates Using Original Seed Stocks or Modern Diploid Progenitor Cell Types

Despite the high sustainability and stability of the original diploid cell banks established in the 1960s, specific technical problems have arisen as even the best cryopreservation storage practices have not made the preserved vial impervious to the effects of time. With the example of the MRC-5 cell source, the importance of the historic use of such cells has been brought to the attention of the World Health Organization (WHO) for sustainable exploitation of the remaining materials. Therefore, the original PDL 7 stock (i.e., population doubling level 7) established in 1966 was recently renewed over concerns of deteriorating quality of original glass ampoules and related stability or biosafety risks [22]. The MRC-5 PDL 13 seed bank was therefore established by the National Institute for Biological Standards and Control (NIBSC) and considered for homologation as a WHO reference cell bank in order to be able to sustainably (i.e., during several decades) provide substrates for vaccine product development and manufacture (i.e., in particular for validation and testing phases) [53]. Materials may be provided directly to individual manufacturers, which bare the responsibility of developing and qualifying specific master cell banks (MCB) and working cell banks (WCB) for their own use. 

**Figure 4:**
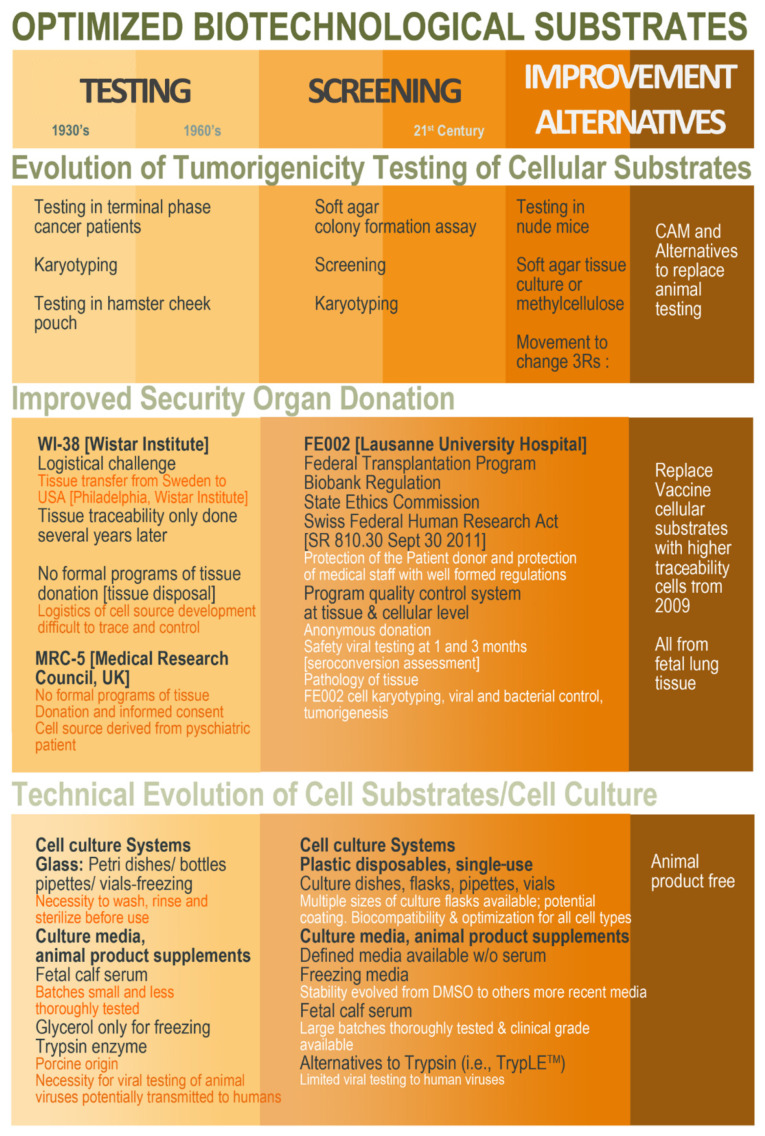
Schematic overview of the ethical, legal, and technical parameter evolution around the establishment of cell sources to be characterized and qualified for use as vaccine production substrates. Overall, hindsight and experience gathered from historic industrial practice may be conjugated with modern regulatory and technical workflows in order to develop optimized (i.e., safe, sustainable, and efficient) biotechnological substrates obtained in modern regulatory settings and methodological workflows. CAM, chorioallantoic membrane; DMSO, dimethyl sulfoxide.

In parallel to the renewal of seed stocks of existing cell types, current efforts are also allocated toward the establishment of novel cell types within modern legal workflows and technical capabilities [45,46,47,48,50]. The objective would be to develop more extensively traced cell sources, with the development of a seed stock or parental cell bank (PCB) at very early passage levels, in order to increase overall security by using modern cell culture and testing technologies, as well as validated methodological workflows (Figure 4).

Despite numerous advantages of such modern approaches (e.g., full safety assessment, documentation of donor consent, use of universal cell stocks), high inertia exists within manufacturer and regulatory circles with regard to the homologation of novel cell types. A noteworthy example of modern diploid cell type establishment is the Chinese Walvax-2, derived from prenatal lung tissue and proposed in 2015 (Wuhan, China) as a qualified substrate for viral material propagation [20]. The need for high quantities of cell substrates to satisfy the Chinese industrial demand, along with restrictions on the availability of imported MRC-5 cells, prompted the development of this new diploid cell source. Specifically, the Chinese Pharmacopoeia (Volume III, 2010) limits the use (i.e., for vaccine manufacturing) of human diploid cell strains to two-thirds of the qualified in vitro lifespan, rendering the dependency on imported cell sources intolerable in terms of supply chain risk management [20]. 

For the isolation and establishment of the Walvax-2 cell source, the original methods of Hayflick (i.e., 1:2 dilution for cell culture procedures) were replicated [1,20]. Although this procedure enables the development of large stocks of cells, it is possible to further maximize production with the implementation of optimized technical specifications, which would be of high interest for extensive up-scaling needs [25].

**Figure 5:**
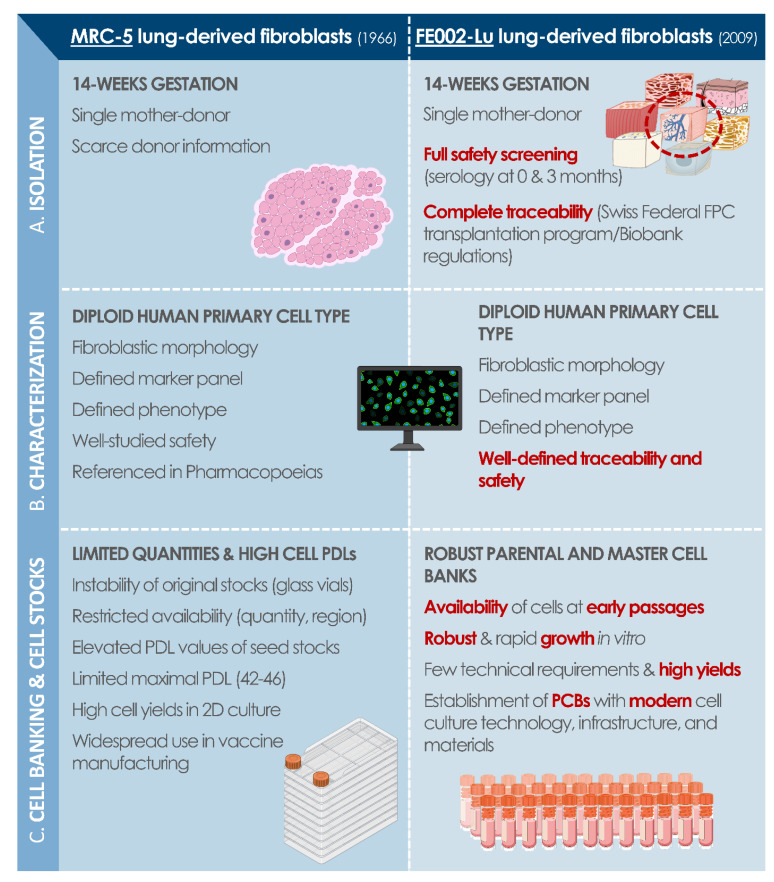
Modern cell source replacement potential, with a comparative assessment of MRC-5 and FE002-Lu diploid cell types, which were both established following cell isolation from prenatal lung tissue (i.e., 14 weeks of gestation). (**A**) Isolation of primary cells from donated tissue samples for initiation of in vitro culture. (**B**) Characterization data related to established cell type quality attributes. (**C**) Technical overview of the similarities and differences between both considered cell sources in terms of cell culture and cell banking. FPC, fibroblast progenitor cells; PCB, parental cell bank; PDL, population doubling level.

Overall, vast opportunities for vaccine substrate renewal exist and have been underlined by current shortages, themselves exacerbated by the COVID-19 pandemic. Novel diploid cell sources have been developed, similar to historically used materials, with prenatal and perinatal tissues (e.g., Walvax-2 diploid human lung cells, CCRC-1 hUC-MSCs, and diploid human amniocytes). In particular, based on the large available data on human diploid progenitor cell GMP banking in Switzerland for therapeutic material sourcing, high interest is currently set locally on specific cell types (i.e., FE002-Lu lung diploid progenitor cell types) for candidacy as novel and robust cell source establishment [25]. Indeed, specific requirements for biological material sourcing, cell type establishment, and cell banking have already been met for FE002 diploid cell types for use thereof as active pharmaceutical ingredients, showing strong overlaps with the requirements set forth for vaccine substrates [31,32,33,42,43,44,45,55,56,57]. To illustrate these parallels, a comparative analysis of MRC-5 cells and FE002-Lu cells was performed and summarized (Figure 5). In particular, modern methodological and technical approaches to original cell sourcing appear as critical and key points for justification of regulatory compliance of considered modern human diploid cell sources. 

## 6. Critical Methodological Aspects of Modern Cell Type Sourcing from Organ Donations and Cell Source Establishment

Organ donations have become central in many advances to medicine and are therefore regulated under strictly defined programs. In Switzerland, the specific legal framework in transplantation medicine is of potentially high interest to devise programs that clearly define procurement and usage of human tissues for establishing primary diploid cell sources (Table 2).

The Swiss Constitution provides that the Federal Council may fully elaborate laws concerning research on human beings (i.e., art 118b Cst) and transplantation medicine (i.e., art 119a Cst). In Switzerland, women may undergo voluntary interruptions of pregnancy without being penalized by criminal law until twelve weeks (i.e., post-amenorrhea, art. 118 al. 3. Swiss Criminal Code, SR 311.0) and at later stages of gestation if attested by a medical doctor in the possibility of physical danger or deep distress (i.e., art. 119 al. 1. Swiss Criminal Code) [30]. Importantly, art. 35 of the Ordinance of the Transplantation Act sets strict measures and guidelines stating that tissues or cells from a prenatal organ donation may be requested only after the mother-donor has made a confirmed decision for gestation interruption [30]. Clear and exhaustive information must be discussed and formalized in writing concerning the aim and nature of the intended use of tissues and cells, as well as the nature and extent of testing to be performed in view of ensuring the quality and safety of considered biological materials. In addition, a reasonable period of reflection is thereafter provided to confirm full consent and understanding. Full compartmentalization and independence between medical staff involved with the patient and the staff involved in tissue bioprocessing and cell transplantation must be assured, and the latter may not have any direct interaction with the donor (i.e., art. 41 Transplantation Act) [30]. 

**Figure 6:**
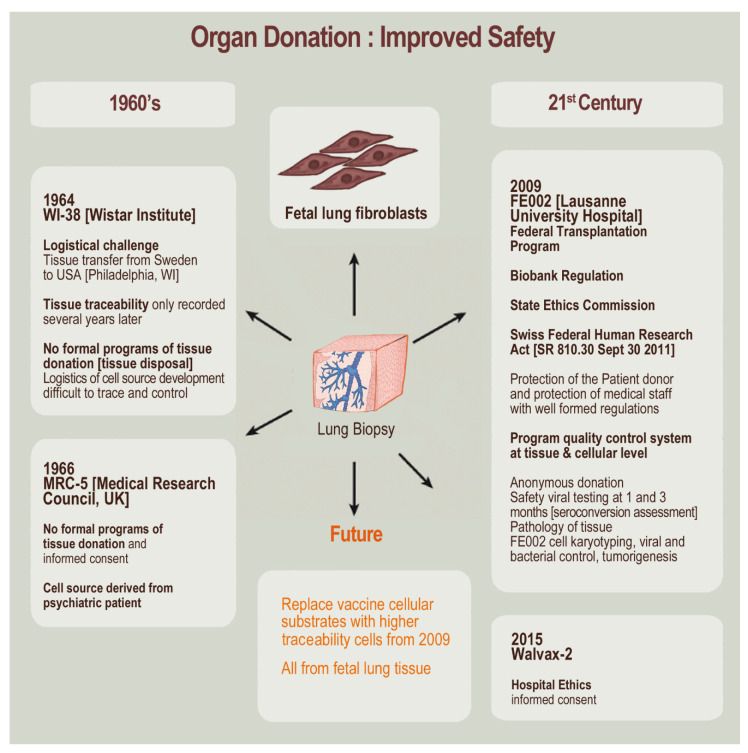
Improved safety of organ donations for diploid cell source establishment with the evolution of practices between the 1960s and the 21st century. Prenatal lung tissue-derived diploid cell sources have been employed as major substrates for vaccine production since the 1960s. The first source was developed in 1964 (i.e., WI-38 cell type), and a later source was developed with better tracing and processing of tissue in 1966 (i.e., MRC-5 cell type). Based on historical practices, comparable modern cell sources were developed between 2009 and 2015 (e.g., FE002-Lu and Walvax-2 human diploid cell types) with enhanced traceability and additional safety features related to ethics and regulatory oversight.

Specific safety procedures are then established, specifying sequential serological testing of the donor at the time of donation and after an appropriate period in order to exclude sero-conversion for specified pathogens. During this period (i.e., ≥3 months), the established cryopreserved cell stocks are quarantined, as out-of-specification test results from the repeated pathogen screening or retraction of the donor with regard to the inclusion of materials in the transplantation program would warrant the destruction of established stocks. As regards ethical and legal oversight, the devising and execution of the transplantation program must be submitted to appropriate controls (e.g., State Ethics Commissions) and defined in framework documents (e.g., Biobank Regulations). Therefore, it is important to note several major differences (i.e., when comparing the 1960s period and present times) in processes and regulations for human research, ethical issues, and informed consent obtention in the context of diploid cell sourcing (Figure 6).

The evolution pertaining to organ donation traceability and safety since the 1960s demonstrates the importance of tissue sourcing and specifically for fully informed consent obtention within applicable legal frameworks. Specifically devised transplantation programs and biobanks assure that material and information traceability is ensured, with appropriate ethical review and oversight levels, and that full informed consent is available (i.e., including for the potential development of therapeutic and/or commercial products with the considered biological starting material). For optimal technology development and overall quality assurance, interdisciplinary approaches for organ donation and processing are warranted. Lawyers may help to interpret regulatory issues of organ donations and biological material use; biologists may select the appropriate cell source and process technical specifications; bioengineers may choose delivery and tissue engineering designs or preservation conditions; medical doctors may perform donor screening and informed consent documentation [31].

## 7. Safe Clinical Experience around the Use of Skin Diploid Progenitor Cells as Active Pharmaceutical Ingredients in Cutaneous Regenerative Medicine

Various musculoskeletal and related soft-tissue diploid progenitor cell types have been studied over the past two decades, and dermal cells (i.e., FE002-SK2 cell types) have been successfully implemented in clinical use [25,26,33]. Most of the local experience has been generated around cutaneous tissue reconstruction and/or regeneration, with progenitor cells being specifically applied as an API for the management of pediatric burns (i.e., second-degree thermal wounds and related graft donor-sites) and acute or chronic inflammatory cutaneous wounds (i.e., lacerations and refractory geriatric lower-limb ulcers) [36,39,41,43,44]. To treat these skin conditions and wounds over the past twenty years, viable progenitor cells were formulated in collagen scaffolds to form progenitor biological bandages (PBB). Such constructs have been implemented as temporary skin coverages for autograft donor-sites and second-degree thermal cutaneous wounds for optimal tissular repair or regeneration promotion. Clinical applications of PBBs have been performed in the Lausanne Burn Center, where growing experience around the use of such products is gathered by a multidisciplinary team. 

The most notable effects of PBB applications have been the reduced tissue scarring following recovery, with excellent structural and functional skin barrier restoration [33,44]. Additionally, reduced pain and inflammation have been observed upon application of PBBs, albeit without standardized and controlled documentation [44]. Therefore, based on translational experience around such biologicals, cell therapy approaches for the management of inflammatory lung diseases or affections and related symptoms (e.g., COVID-19 patients and burn patient severe inhalation syndrome) using local homologous allogeneic cell-based or cell-derived product administrations are considered. Specifically, prenatal lung tissue-derived diploid progenitor cells (e.g., FE002-Lu cell type) appear as optimal candidates for the development of an API in such regenerative medicine applications. For practical design and prototype development of diploid progenitor cell-based products for lung tissue treatment, many aspects may be transposed from the field of stem cells, which have been considered and investigated extensively for wide arrays of applications [25]. Specific aspects of the delivery vehicle and delivery route selection, cell dosing regimens, and the optimization of product effects may also be inspired by previous studies around cutaneous tissue regeneration, yet specificities of the target lung tissues and related pulmonary affections require adaptations. 

## 8. Multi-Tiered Cell Banking of Diploid Lung Tissue-Derived Progenitors

Various technical methodologies for lung tissue-derived progenitor cell isolation and culture initiation may be adopted (e.g., mechanical dissociation or trypsin-based tissue digestion), whereas the official context or framework for tissue sourcing and procurement should be adequately defined (e.g., transplantation programs), as described previously [25]. 

Following strict procedures and controlled access to materials and information, prenatal lung tissue samples are anonymously yet traceably obtained for subsequent bioprocessing, ensuring optimal quality and safety of progeny cell sources. Technical specificities of lung progenitors place such cell sources at the forefront of potential therapeutic material candidates for regenerative medicine applications. Indeed, rapid establishment and extensive cell banking capabilities practically negate the need for repeated organ donations, as attested by the industrial use of the MRC-5 cell type, for example, which has lasted over fifty years thus far following a single tissue donation [16]. Therefore, repeat testing and validation of new cell sources or pooling thereof are not required, and this aspect contributes to augment safety and to lower overall manufacturing costs. With regard to technical aspects of primary lung progenitor cell isolation, parental cell banks (PCB) at early passages may be rapidly established, composed of several dozen vials, each containing 10^6^ to 10^7^ cells to be preserved [32]. Standard culture media (e.g., DMEM supplemented with fetal bovine serum) and culture conditions (e.g., humidified atmosphere under 5% CO_2_ and 37 °C incubation) are sufficient for initiation of adherent lung tissue-derived progenitor cell cultures in vitro. 

**Figure 7:**
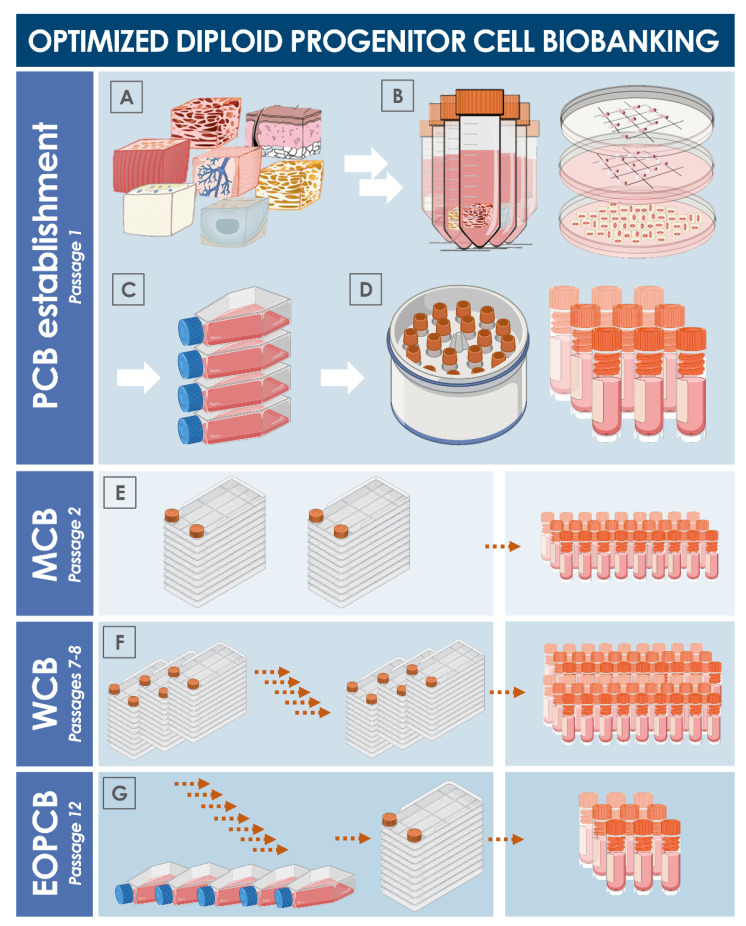
Schematic overview of the technical steps to be undertaken for primary diploid cell type establishment and serial culture expansion for multi-tiered cell bank establishment. (**A**) Procurement of specific starting biological materials. (**B**) Enzymatic or mechanical cell dissociation. (**C**) Preliminary expansion in cell culture vessels. (**D**) Cryopreservation and constitution of a parental cell bank. (**E**) Manufacture of master cell banks. (**F**) Manufacture of working cell banks. (**G**) Manufacture of an end of production cell bank. Exhaustive documentation of the successive steps and thorough characterization, qualification, and release testing schemes allow for the safe and sustainable use of considered diploid cell sources. EOPCB, end of production cell bank; MCB, master cell bank; PCB, parental cell bank; WCB, working cell bank.

Due to the high robustness and extensive proliferative potential of primary progenitor cells, such sources may be used in industrial-scale cell banking campaigns under good manufacturing practice (GMP) requirements [41]. For an optimal and sustainable use of progenitor cell biobanks, serial expansions may be performed for the eventual establishment of multi-tiered cryopreserved stocks of cells (Figure 7). Inherent characteristics of diploid progenitor cell types enable thorough iterative testing and validation steps to be performed on all cell bank tiers, which may be allocated into parental cell banks (PCB), master cell banks (MCB), and working cell banks (WCB). Additionally, end of production cell banks (EOPCB) may be generated for technical and safety qualification of the cell types of interest. Culture reagents and conditions described hereabove for primary cell isolation remain the same for subsequent expansions, yet extensive optimization must be carried out in a pilot cell banking campaign to define, among other parameters, the optimal culture vessels (i.e., type, model, surface, oxygenation method), media supplement source (i.e., fetal bovine serum supplier and lot number), and culture maintenance workflows (i.e., culture medium volumes, medium exchange rates, cell seeding densities, cell harvesting confluency, culture period duration). Once optimal technical specifications have been established, large-scale cell banking may be tangibly performed. Stringent in-process controls and material testing must be adapted for quality assurance purposes. At each step of the cell banking process, adequate product characterization and release testing must be performed on manufacturing lots (e.g., recovery assays, isoenzyme testing and DNA fingerprinting, sterility testing, or research of microorganisms with a particular focus set of viruses of human, bovine, and porcine origin). Additional testing performed on EOPCB materials (e.g., in vitro and in vivo tumorigenicity assays, karyology studies) enable the qualification of the safety and stability of considered progenitor cell types [77,78,79,80,81]. 

## 9. Potential Therapeutic Applications of Diploid Lung Progenitors in Respiratory Tract Regenerative Medicine

**Figure 8:**
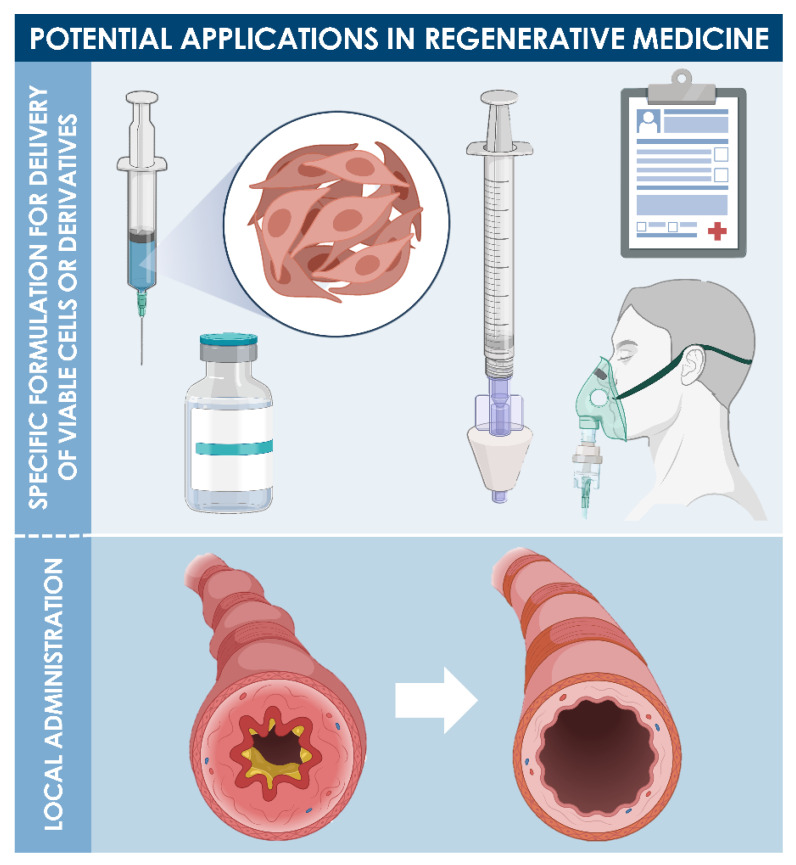
Potential applications and postulated effects of diploid progenitor cells in respiratory tract regenerative medicine. Appropriate cell-based product formulations are considered for local delivery in view of obtaining potential anti-inflammatory or tissue modulation effects during recovery. The overall goal of local product administrations would be to limit airway inflammation and reduce tissue scarring upon recovery for optimal structural and functional lung tissue restoration.

Cultured diploid progenitor cells may be considered as optimal biological sources presenting potentially vast therapeutic utility in the domain of inflammatory lung diseases and control of symptoms thereof (Figure 8). Due to the current extreme numbers of COVID-19 patients around the world, a specific focus has been set on the use of cell therapies and derived biologicals for enhanced management options in clinical workflows. Diverse stem cells and perinatal cells, in particular, have been proposed as candidates and have been the objects of multiple clinical trials (e.g., ClinicalTrials.gov identifiers: NCT04333368, France; NCT04313322, Saudi Arabia; NCT04315987, Brazil; and ChiCTR2000029990, China) [82,83,84,85,86,87,88,89,90,91,92]. 

Therefore, similar to other domains of regenerative medicine, the origin and processing of the considered therapeutic cellular materials are often limiting factors in the effective development of novel therapies or products, mainly due to safety, consistency, sustainability, or manufacturing cost issues [93,94,95,96,97,98,99]. Adequately isolated lung tissue-derived progenitors (e.g., FE002-Lu cell types) constitute prime developmental candidates due to their tissue-specific origin, high consistency, and high stability [25]. These aspects may be used for the comparison of various cell sources proposed as APIs for respiratory tract regenerative medicine (e.g., progenitor versus stem cells). Indeed, several quality, consistency, and sustainability issues exist around the use of stem cells for lung delivery (e.g., safety, survival, availability in sufficient quantities) [82,85]. Technical problems, such as high individual treatment doses (e.g., several million cells/kg) and treatment administration logistics have been reported, limiting the easy and widespread use of such approaches [83,84]. Noteworthy alternatives yet parallel approaches to stem or progenitor cell therapy consist of the functional restoration of normal resident lung progenitor cells (e.g., ATII cells) using various approaches or the therapeutic use of induced pluripotent stem cell (iPSC) technology for the management of diverse lung injuries (e.g., caused by chemical aggression or hyperoxia) [100,101,102,103].

Based on the vast translational experience with skin-derived progenitors and their various putative in vivo therapeutic modulatory effects, a similar homologous cell therapy approach could be employed using lung tissue-derived progenitors for the local management of inflammatory lung diseases and related symptoms (Figure 8). Various administration routes exist for managing inflammatory lung diseases, among which intravenous infusions, intra-tracheal administration, and local delivery via nebulizers, for example, which, respectively, define the formulation processes that will be applied to therapeutic cellular APIs or derivatives [83,84,85]. The first option (i.e., therapeutic cell infusions) takes advantage of the fact that the lungs constitute a major bottleneck in the pharmacokinetics (i.e., major biodistribution reservoir) of such cell therapies, wherein the therapeutic API is administered in relatively large doses and is naturally distributed to the intended target tissue [87]. The second and third delivery options (i.e., local delivery via injection or aerosolization, for example) may be more interesting for a number of reasons, among which a lower overall product dose, relatively easier product administration, and localized effects [85,86].

Due to the fact that human therapeutic lung tissue-derived progenitors have undergone substantial manipulation (i.e., in vitro culture expansions during manufacture), final cell-based products may be considered as standardized transplants and/or (combined) advanced therapy medicinal products (ATMP) from a regulatory point of view, depending on the specific formulation and processing applied [63,64,65]. If devitalized cellular materials, cellular derivatives, or cellular byproducts are used instead of viable lung progenitors, the regulatory classification may differ depending on the country and region, yet current requirements for product registration progressively decrease the attractiveness of alternative classification routes. Furthermore, in the context of unified efforts against the COVID-19 pandemic, numerous incentives and opportunities have been created with regard to the registration of novel biologicals and cell therapies, as attested by the high number of ongoing clinical trials, as mentioned previously [82]. 

The exact mechanism of action of therapeutic progenitors has not yet been exactly elucidated but probably relies on some form of synergistic paracrine or trophic modulation of patient target tissues by multiple cellular components (e.g., structural proteins, soluble factors, enzymes) [41]. Based on the observed effects of skin-derived progenitors on cutaneous burn wounds and surrounding tissues, it was hypothesized that such cells mainly exert their therapeutic effects by modulating tissular inflammation, immune reactions, and resident cell proliferation or migration [41]. In particular, skin-derived progenitors were shown to promote the resurgence of optimal skin structure and function after burn wound closure, with lowered long-term complication rates and less formation of fibrotic scar tissue [44]. If such effects and results can be transposed to affected lung tissues (e.g., inhalation injuries in burn victims or inflammation states following viral infections) using lung tissue-derived progenitors, several short-term and long-term benefits may be potentially gained for treated patients, with a reduction in inflammatory symptoms and optimal healing of lung tissues, respectively.

Therefore, further studies are necessary in preclinical and clinical settings to better characterize the in vivo effects of therapeutic lung-derived progenitors or derivatives thereof, as abundant research in the neighboring field of stem cells has evidenced preponderant roles of cell secretomes or sub-cellular vesicles in mechanisms of repair and/or regeneration [88,89]. Such considerations may further direct the developments of lung-derived progenitor-based products, as cell-based cell-free versions present several logistical, technical, regulatory, and safety advantages. 

## 10. Potential Pathways for Diploid Lung Progenitor Cell Type Homologation as an API

In addition to the qualification of modern lung tissue-derived progenitor cell sources as vaccine substrates, optimization of regulatory aspects of potential direct therapeutic uses thereof is of equal high interest. In this sense, homologation of the cell source in an appropriate form would enhance the transparency and safety levels pertaining to the API of considered investigational medicinal products. From a technical standpoint, the use of standardized progenitor cells or cell derivatives as APIs has significant advantages, notably in terms of traceability and consistency. Due to the extensive expansion potential of selected primary cells, an individual cell type may be thoroughly tested and fully qualified in terms of safety, similarly to MRC-5 or WI-38 cells, which are recognized as safe (i.e., non-tumorigenic) by pharmacopeias, without the necessity for repeated testing [57]. This aspect contrasts with the mandatory iterative testing workflows relative to pooled batches of primary donor cells, implemented for biological materials which cannot be originally isolated in sufficient quantities or cannot be expanded sufficiently to be independent from renewed tissue donations [50]. From a material consistency and sustainability standpoint, the centralization of cell deposits for further multicentric use would enable a facilitated implementation of standardized protocols for cell manufacture. Overall, this would help to ensure traceability of the original material and mitigation of potential related risks (i.e., assurance of ethical and legal exposure absence), with additional simplification of the documentary workloads for manufacturers (i.e., identical cell type master file and technical specifications for manufacturing).

With regard to cell source homologation or optimization of regulatory statuses, three pathways are considered herein, namely the creation of pharmacopeial monographs (i.e., general or specific), the creation of a cell and tissue monograph, or the registration of specific cell sources as WHO reference cell banks. Firstly, the inclusion of a cell monograph in a pharmacopeia at a supranational level (e.g., European Pharmacopoeia for the whole European Community) would enable the facilitated prescription of cell-based preparations in hospital settings, for example, with the setting of adequate technical and quality specifications. A recent example of a cell monograph introduced in the European Pharmacopoeia is the monograph 2323, “Human hematopoietic stem cells”, which may serve as a basis for the elaboration of adequate texts relative to lung tissue-derived progenitor cells of appropriate quality. Secondly, a cell and tissue monograph may be considered, as set forth in the “Guide to the quality and safety of tissues and cells for human application” of the EDQM, with a similar definition of technical specifications and quality attributes of considered cell APIs or products, once the clinical experience is gathered [50]. Thirdly, the proposal of a specific cell source for recognition as a WHO reference cell bank would constitute an optimal pathway for the sustainable and multicentric use of a homogenous cell type for vaccine manufacturing and/or regenerative medicine product API production. This could potentially be explored via collaborations with experts and the WHO Expert Committee on Biological Standardization [22,53,61]. 

Specifically, the criteria to be met for the possible inclusion of cell banks in the WHO reference cell bank system are presented in Table 3. Therefore, further discussion and collaboration with national or supranational bodies and organizations are necessary in order to tangibly attain some specific form of homologation for the biological materials of interest.

## 11. Conclusions

Based on the technical specificities of primary diploid progenitor cell types and the historic experience around the safe industrial and clinical uses, it is currently of high interest to further investigate lung tissue-derived progenitor cell sources for developing novel cell therapy products or biotechnological substrates. The high demand for adequate and renewed cell substrates was illustrated with the current pandemic context and the extensive needs of the vaccine industry. Furthermore, increasing numbers of patients presenting inflammatory or degenerative lung tissue affections prompt the development of novel and effective therapeutic products, among which cell-based or cell-derived solutions. Overall, it is expected that lung tissue-derived progenitors produced under good manufacturing practices may be used with high versatility, either as key industrial platforms conforming to specific pharmacopeial requirements or as active pharmaceutical ingredients in advanced therapy medicinal products or standardized transplants. Both approaches are highly interesting and potentially valuable in current and continued struggles against the COVID-19 pandemic, with potential contributions to industrial development efforts and tangible support in global clinical challenges around patient therapeutic management.

## Figures and Tables

**Table 1:** Summary of main technical rationale aspects related to the development and adoption of primary human diploid cell sources, as set forth in the 1960s and developed during the search for optimal viral propagation tools. Identical technical rationale applies to recently established progenitor diploid cell sources to be used in regenerative medicine products and biotechnological manufacturing workflows [1,2,53]. PDL, population doubling level; PL, passage level.

Technical possibility to cryopreserve extensive homogenous cell lots at relatively early population doubling levels (PDL) or passage levels (PL) within the qualified in vitro cell type lifespan.
Original establishment of appropriate cell types and characterization of the derived cryopreserved cell banks, which may later be used as starting materials for further multi-tiered banking and sustainable provision of standardized cell sources for research and industrial applications.
Implementation of extensive and appropriate (i.e., risk analysis-based) biosafety testing schemes for the qualification of manufacturer-specific cell banks before use in vaccine production activities.
Demonstration that the considered cellular materials are exempt from detectable adventitious agents and that they are unable to form tumors when inoculated into immunosuppressed animals. ^1^

^1^ Drastic evolution of methodologies for safety testing occurred, with the successive updates of specific regulations on research conducted on human subjects (e.g., original tumorigenicity testing carried out on terminal cancer patients) [1].

**Table 2:** Legal and regulatory frameworks involved in modern procurement of tissues for primary diploid cell type establishment and use in Switzerland. EU, European Union.

Framework Document	Jurisdictional Level and Application
Human research and consent	Regulated in federal laws and applicable EU texts
Federal Transplantation Program ^1^	Registered with the Federal Office of Public Health or the Swiss Institute for Therapeutic Products (Swissmedic)
Ethics Commission	Regulated at a state level by the Ethics Committees
Biobank Regulations ^2^	Regulated at an institutional level in University Hospitals or within the framework of a local/regional/national biobank system

^1^ Assures anonymous status and no financial gain for the organ/tissue donation. ^2^ Informed consent (i.e., full transparency if tissue or cells will be used to produce therapeutic and/or commercial products).

**Table 3:** Summary of the various criteria to be met by specific cell types or sources in view of proposition thereof as WHO reference cell banks. Such cells banks are specifically designed to offer potential solutions to emerging challenges in the development of vaccines and biotherapeutics. The specific technical purpose of such cell banks is to be able to sustainably provide medicinal product manufacturers with well-characterized cell seed materials to be used for establishing manufacturer MCBs, to be further characterized and appropriately qualified [53]. MCB, master cell bank; WHO, World Health Organization.

• Full traceability is available to the origin of the cell source, around derivation of the cell line/type, and materials used in the preparation of the cell seed stocks.
• The research is subject to open international scientific scrutiny and collaborative technical investigations into the characteristics of the cells and the possible presence of adventitious agents.
• The cell characterization results are peer-reviewed and published.
• Investigations are evaluated under the auspices of WHO expert review and cells are qualified as suitable for industrial use.
• The supply of cells is free of any constraint related to intellectual property rights on final products.
• A single source of cells exists with a growing, scientifically, and technically updated body of safety-testing data and safe history of use, giving increased confidence to manufacturers, regulators, and public policy makers.

## Data Availability

Not applicable.

## Abbreviations

API	active pharmaceutical ingredient
ATCC	American type culture collection
ATMP	advanced therapy medicinal product
bp	base pairs
CAM	chorioallantoic membrane
CHUV	Centre hospitalier universitaire Vaudois
DMEM	Dulbecco’s modified Eagle medium
DMSO	dimethyl sulfoxide
DNA	deoxyribonucleic acid
EDQM	European Directorate for the Quality of Medicines & Healthcare
EOPCB	end of production cell bank
EP	European Pharmacopoeia
EU	European Union
FBS	fetal bovine serum
FDA	US Food and Drug Administration
FPC	fibroblast progenitor cell
GMP	good manufacturing practices
HIV	human immunodeficiency virus
hPL	human platelet lysate
ICH	International Council for Harmonization of Technical Requirements for Pharmaceuticals for Human Use
iPSC	induced pluripotent stem cells
MCB	master cell bank
MRC	Medical Research Council
MSC	mesenchymal stem cells
NA	not applicable
NIBSC	National Institute for Biological Standards and Control
NIH	National Institutes of Health
PBB	Progenitor Biological Bandage
PCB	parental cell bank
PDL	population doubling level
PL	passage level
SV40	simian virus 40
US	United States of America
WCB	working cell bank
WHO	World Health Organization
WI	Wistar Institute
